# Tree-ring widths are good proxies of annual variation in forest productivity in temperate forests

**DOI:** 10.1038/s41598-017-02022-6

**Published:** 2017-05-16

**Authors:** Kai Xu, Xiangping Wang, Penghong Liang, Hailong An, Han Sun, Wei Han, Qiaoyan Li

**Affiliations:** Key Laboratory for Forest Resources & Ecosystem Processes of Beijing, and College of Forestry, Beijing Forestry University, Beijing, 100083 China

## Abstract

Tree rings have long been used to calibrate the net primary production (NPP) time-series predicted by process-based models, based on an implicit assumption that ring-width indices (RWI) can well reflect temporal NPP change. However, this assumption has seldom been tested systematically. In this study, 36 plots were set in three forest types from four sites along a latitudinal gradient in northeast China. For each plot, we constructed chronologies and stand NPP of the past 20 years to examine: is RWI a good proxy of inter-annual variation of forest NPP for different forest types under different climate? If it is, why? Our results indicate that RWI was closely related to stand NPP in most cases, and could be used as a good proxy of NPP in temperate forests. Standard and arstan chronologies were better related to NPP series than residual chronology. Stand NPP time-series were mainly determined by large trees, and the correlation between RWI and NPP was also higher for larger trees. We suggest that large trees and dominant species of canopy layer should be sampled for chronology construction. Large trees are major contributors of forest biomass and productivity, and should have priority in forest conservation in a rapid-warming world.

## Introduction

Net primary production (NPP) is a key component of terrestrial carbon cycle, and the response of NPP to climate change has long been a focus in ecology^1, 2^. However, mapping and monitoring forest NPP at large to global scale is extremely hard through field-measurements. Consequently, ecological modeling has become an indispensable tool to estimate large-scale patterns of NPP and carbon budget. Process-based modeling has gained rapid progresses in the past decades, because it provides powerful tools to predict response of ecosystems productivity to future climate change^3–7^.

However, process-based modeling has long been puzzled by the lack of field-measured data for model validation^8^. Recent studies have increasingly stressed the necessity to calibrate the models for both their predictions on temporal variation and spatial patterns (e.g. refs 9 and 10). Nevertheless, the lack of long-term observations has prevented many studies from validating the time-series predicted by their models. Generally, three types of data have been used for model validation: (1) repeated measurements of permanent plots (e.g. ref. 11); (2) carbon flux data estimated from the eddy covariance method (e.g. ref. 12); (3) tree ring widths data. However, the time interval to revisit permanent plots is typically several years. Thus the temporal resolution is not enough to tested the models’ ability to simulate the inter-annual variability of NPP, which was found to be critical for model validation^9^. Similar as permanent plots, flux tower data are also only available for a limited number of sites. Meanwhile, flux tower data are often of short duration (despite high temporal resolution). For instance, there are only six flux towers across northeast China’s forests (which cover an area of 47.0 × 104 km^2^), with most of the towers established only recently and located at lower altitudes^13^. For forests at high altitudes, which are suggested to be more sensitive to climatic change^14, 15^, no flux tower data are available in this region.

On the other hand, tree rings are widely available from remote sites, and have the advantage that both the temporal resolution and time duration are good enough. Consequently, ring width index (RWI) have long been used for validating biogeochemistry models (e.g. refs 16–19). However, RWI is an index of annual radial growth at the scale of individual trees^20^, while stand NPP is a measurement at community scale. Thus, using RWI to validate modeled NPP time-series is actually based on an implicit assumption that RWI can well reflect the temporal change of stand NPP. However, this assumption has seldom been tested systematically, despite that a number of studies have used RWI for model validation. It should be noted that, until now, how much of stand NPP is allocated to annual DBH (diameter at breast height) increment is still not well understood because it depends on a number of biotic and abiotic factors^17, 21^. This poses a key challenge to utilizing tree rings for the calibration of biogeochemistry models. Consequently, the first aim of our study is to test whether RWI is a good proxy of annual change in stand NPP, for different forest types under different climate. At the same time, three kinds of chronologies are generally constructed in dendrochronology (standard, residual and arstan chronology). Until now, it is still unknown which chronology is a better surrogate of stand NPP series (and thus should be adopted in model validation). Our second aim is to examine this question.

If our tests show that RWI can be used as a surrogate of NPP time-series, then an interesting question is raised: why RWI can reflect the temporal NPP change at community scale? To examine this question, we tested three predictions based on current knowledge as follows. (1) Recent studies have showed that stand biomass is largely occupied by large canopy trees^22–24^, and it is well known in ecology that individual productivity is closely related to its biomass^25^. Consequently, we predict that stand NPP is also largely determined by the NPP of large trees (**P1**). (2) If P1 is true, then annual stand NPP should be closely related to the annual NPP of large trees. On the other hand, the smaller the trees, their productivity should be more affected by the canopy dynamics of upper-layer trees, but less affected by temporal variation in climate (which is a major cause of the fluctuation of stand NPP). Thus it is predicable that the correlation between stand and tree NPP series should be lower with decreasing tree size (**P2**). (3) In forest communities, the tree-ring cores used for chronology construction are commonly sampled from large trees at canopy layer. This sampling strategy is not only aimed to obtain longer time-series, but also helps to reduce the influence of competition between trees in order to maximize the climate signals in tree ring data. We predict that the chronologies thus obtained should be more closely related with the NPP series of larger trees, but less closely related to that of smaller trees (**P3**). Taking together, we hypothesized that the reason why RWI can be used as proxy for forest NPP dynamic is that stand NPP is predominantly determined by the annual NPP variation of large trees, while the latter is closely related to annual DBH growth of large trees.

In this study, we examined the relationship between ring widths and NPP series in three typical forest types along a latitudinal gradient in northeast China. We asked three questions by follows. (1) Are tree-ring widths good proxies for annual variation of stand NPP, for each forest type in each site? (2) Which type of tree-ring chronology is a better surrogate of stand NPP? Standard, residual or arstan chronology? (3) Why ring-width series can reflect the temporal dynamic of NPP at stand level?

## Materials and Methods

### Study sites

To test whether RWI is a good proxy for annual variability of stand NPP, for different forest types under different climate, we selected four sites in northeast China (Table 1). The study sites covered a latitudinal gradient from 42.3°N to 49.5°N, with annual mean temperature ranged between −2.1 °C and 2.6 °C, and annual precipitation between 510 and 810 mm. In each site we sampled three typical forest types in northeast China: (1) *Betula* & *Populus* forest (BPF), dominated by *Betula platyphylla* and *Populus davidiana*; (2) deciduous broad-leaved forest (DBF), composed of *Quercus mongolica*, *Tilia* spp., *Acer* spp. and *Fraxinus mandschurica* etc.; and (3) mixed broad- & needle-leaved forest (MBNF), composed of *Pinus koraiensis* and broadleaf species such as *Fraxinus mandschurica*, *Tilia* spp., *Acer* spp. etc. We established three plots (50 × 20 m) as replicates in each forest type by each site, and a total of 36 plots were investigated in the summer of 2012 (Mt. Changbai and Jiaohe) and 2013 (the Wuying and Shengshan sites). In each plot, we recorded geographic coordinates (latitude, longitude and elevation), each tree with a DBH > 3 cm was identified to species and the DBH at 1.3 m was measured.

**Table 1:** General information of four study sites in Northeast China.

Site	Latitude (°N)	Longitude (°E)	Altitude (m)	AP (mm)	AMT (°C)
Mt. Changbai	42.35	127.82	953	850	1.2
Jiaohe	43.96	127.74	473	700	2.6
Wuying	48.09	129.12	362	510	−0.2
Shengshan	49.50	126.78	512	540	−2.1

Abbreviations: AP, annual precipitation; AMT, annual mean temperature.

### Tree-ring chronology development

In each plot, we selected 30 to 40 large trees to include most of the dominant species in the canopy layer, following commonly used dendrochronological protocols. Two tree-ring cores at breast height were extracted from two vertical directions (north-south and east-west). All cores were dried, mounted and sanded with sandpaper up to 1200 grit until the annual rings can be clearly identified. Then a LINTAB 6 measuring station and TSAPWin software (Frank Rinn Co. Ltd., Germany) were used to measure the ring widths with a resolution of 0.01 mm. The quality of cross-dating and measurement was checked using the COFECHA program^26^ and about 30–40% of initial cores were discarded. For the remaining cores, the growth trends in raw ring-width data were removed with exponential or linear function. Then three chronologies (standard, residual and arstan) were developed for each plot using the ARSTAN program^20^. Standard chronology is developed by first detrending the growth trends associated with tree age in raw ring-width data by different curve fitting methods, and then the standardized ring widths were averaged among samples. Residual chronology further removed the low-frequency growth trend, by using the residuals from autoregressive modeling of individual series, combined using the bi-weight robust mean estimation. Arstan chronology, however, is derived by adding the low-frequency growth trend caused by common persistence among trees back to the residual chronology. For statistical characteristics of these chronologies, see Supplementary Tables S1 and S2. All the chronologies had an expressed population siginal >0.85, suggesting good qualities of these chronologies^27^.

### Estimation of stand NPP series

To estimate the aboveground NPP of each plot, we extracted another set of tree-ring cores for all trees with a DBH ≥10 cm. These cores were short because we wanted to estimate the NPP time-series only for the past 21 years (see below). The cores were sanded and cross-dated, and the ring widths of the past 21 years were measured and checked using the same procedures described above. Then the raw ring-width data were used for NPP estimation.

For estimation of aboveground NPP, we adopted the method used by Graumlich *et al*.^28^. NPP is generally defined as:

1 $$NPP={\rm{\Delta }}B+D+G$$

Where *ΔB* is annual biomass increment, *D* is annual detritus production (litterfall and tree mortality), and *G* is annual herbivore foraging.

In Eq. 1, the loss of production due to foraging is negligible, and the annual fluctuation of litterfall is also negligible for closed-canopy forests^29^. However, estimating the past production losses caused by tree mortality is difficult^28^. Consequently, we estimated only the NPP of past 21 years to minimized the influence of tree mortality. Thus, by assuming that the influences of tree mortality, litterfall and foraging were negligible for temporal NPP variation, annual NPP series were estimated as:

2 $$NPP\approx {\rm{\Delta }}B$$

Using the field-measured DBH and annual ring-width for each tree, the tree’ s DBH in the past 21 years was reconstructed. Aboveground biomass in each year was calculated for each plot by summing the biomass (including leaf, branch and stem) of individual trees, which were estimated by site- and species-specific allometric equations. For plots at Mt. Changbai, we used the equations of Chen and Zhu^30^ and Zhu^31^. For plots at Jiaohe, WuYing and Shengshan we used the equations of Dai^32^, Yang *et al*.^33^ and Hu *et al*.^34^, respectively. In a final step, the aboveground NPPs during the past 21 years for each plot, and for each individual tree, were calculated using Eq. 2. However, the current year NPP (and ring width) were not used in statistical analyses and thus the final NPP and RWI data used were of a 20-year period, because we sampled cores in summer and tree DBH growth may have not finished.

### Statistical analyses

**Figure 1:**
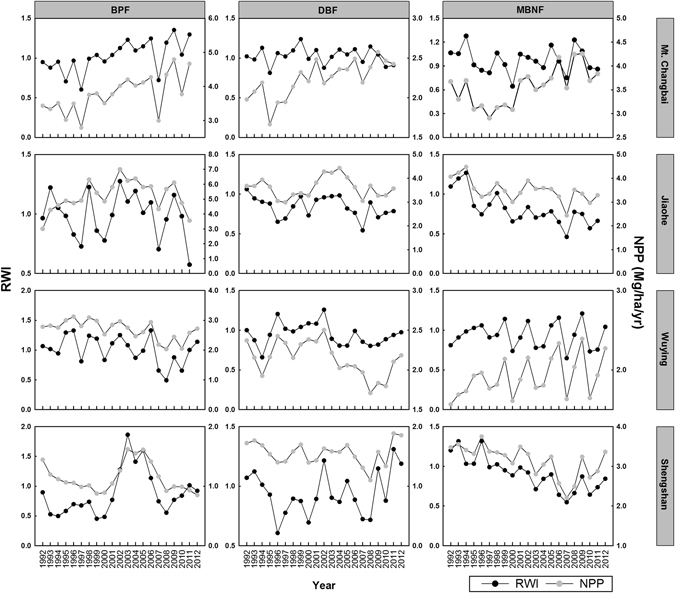
Comparison of temprefd between stand NPP (gray dots, Mg/ha/yr) and standard chronology (RWI, black dots) for each forest type by each site. Abbreviation: BPF, *Betula* & *Populus* forest; DBF, deciduous broad-leaved forest; MBNF: mixed broad- & needle-leaved forest.

If RWI is a good proxy for annual variation of stand NPP (Question 1), then RWI and NPP time-series should be well correlated and they should show similar inter-annual variation. We related the standard, residual and arstan chronologies to NPP series to examine this question, and to explore which chronology is a better surrogate of stand NPP (Question 2). Since the same type of chronology (e.g. standard chronology) were highly correlated among the three replicated plots of each forest type in each site (this is also true for NPP series), in these analyses we averaged the chronologies and NPP-series of the three replicated plots for simplicity in reporting the results (Table 2 and Fig. 1).

**Table 2:** Correlations between stand NPP time-series and tree ring chronologies for three forest types in four sites.

Site	Forest Type	Chronology		
		Residual	Standard	Arstan
Mt. Changbai	BPF	0.940	0.984	0.981
	DBF	*0.289*	*0.360*	*0.348*
	MBNF	0.463	0.573	0.544
Jiaohe	BPF	0.589	0.620	0.621
	DBF	0.526	0.720	0.769
	MBNF	0.732	0.927	0.889
Wuying	BPF	0.781	0.920	0.918
	DBF	0.603	0.828	0.813
	MBNF	0.791	0.839	0.836
Shengshan	BPF	0.460	0.791	0.733
	DBF	0.643	0.810	0.799
	MBNF	0.478	0.912	0.892

All correlations were significant at 0.05 level expect those in *italics*. Abbreviations: BPF, *Betula* & *Populus* forest; DBF, deciduous broad-leaved forest; MBNF: mixed broad- & needle-leaved forest.

To examine why RWI can well reflect the fluctuation of stand NPP (Question 3) we tested three predictions proposed in the Introduction. We grouped trees in each plot into four size classes at the 25%, 50% and 75% quartiles of DBH (so that the tree numbers in the four classes were the same). We summed the NPP time-series for trees in each DBH class. To test our first prediction (P1), for each plot we calculated the proportion of tree NPP in each DBH class to total stand NPP. We predicted that large trees should account for most stand NPP. To test P2, we correlated NPP time-series at plot level to that of each DBH class. We predicted that the correlation should be high for the 1/4 largest trees but decrease with smaller DBH classes. To test P3, in each plot we correlated RWI to the NPP series of each DBH class. We again predicted that the correlation should be the highest for the 1/4 largest trees but lowest for the 1/4 smallest trees.

## Results

### Relationships between stand NPP and RWI

For each forest type in each site, RWI series were significantly correlated with stand NPP, except for the DBF at Mt. Changbai (Table 2). Standard and arstan chronologies were generally better correlated to NPP series than residual chronology, with all *r* > 0.57 and most *r* > 0.75 (not including the one exception). Thus residual chronology seems to be not a good proxy of stand NPP. In the subsequent analyses we reported only the results based on standard chronology because that of arstan were largely the same.

A comparison between RWI and stand NPP series further showed that their annual fluctuations were very similar (except for the DBF at Mt. Changbai) (Fig. 1). Notably, the minimal or maximal values in RWI series (i.e. extremely narrow or wide rings) corresponded well with that in NPP series, suggesting that the years with limiting (or favorite) climate conditions were reflected in both RWI and NPP data. Thus our results suggested that, in most cases, RWI can be used as surrogate of stand NPP series.

### Relationships of NPP in different DBH classes with stand NPP and RWI

The proportion of tree NPP in total stand NPP increased sharply from the smallest (1^st^) to the largest tree group (4^th^) (Table 3). On an average, 84.3% (70.3%~94.6%) of stand NPP was occupied by individuals in the 4^th^ and 3^rd^ DBH classes, supporting our P1 that stand NPP is mainly determined by large trees.

**Table 3:** Summed NPP (means across twenty years) for trees within each DBH class as a proportion of total stand NPP.

DBH Class	Proportion in stand NPP (%)		
	Min	Max	Mean
1st	1.63	11.75	5.48
2nd	3.74	18.94	10.26
3rd	9.81	31.10	21.94
4th	45.83	83.03	62.32

Mean, min and max proportions for the 36 plots were reported here. In each plot, trees were grouped into four quartiles based on their DBH (e.g. 1^st^, the 1/4 trees with the smallest DBHs).

**Figure 2:**
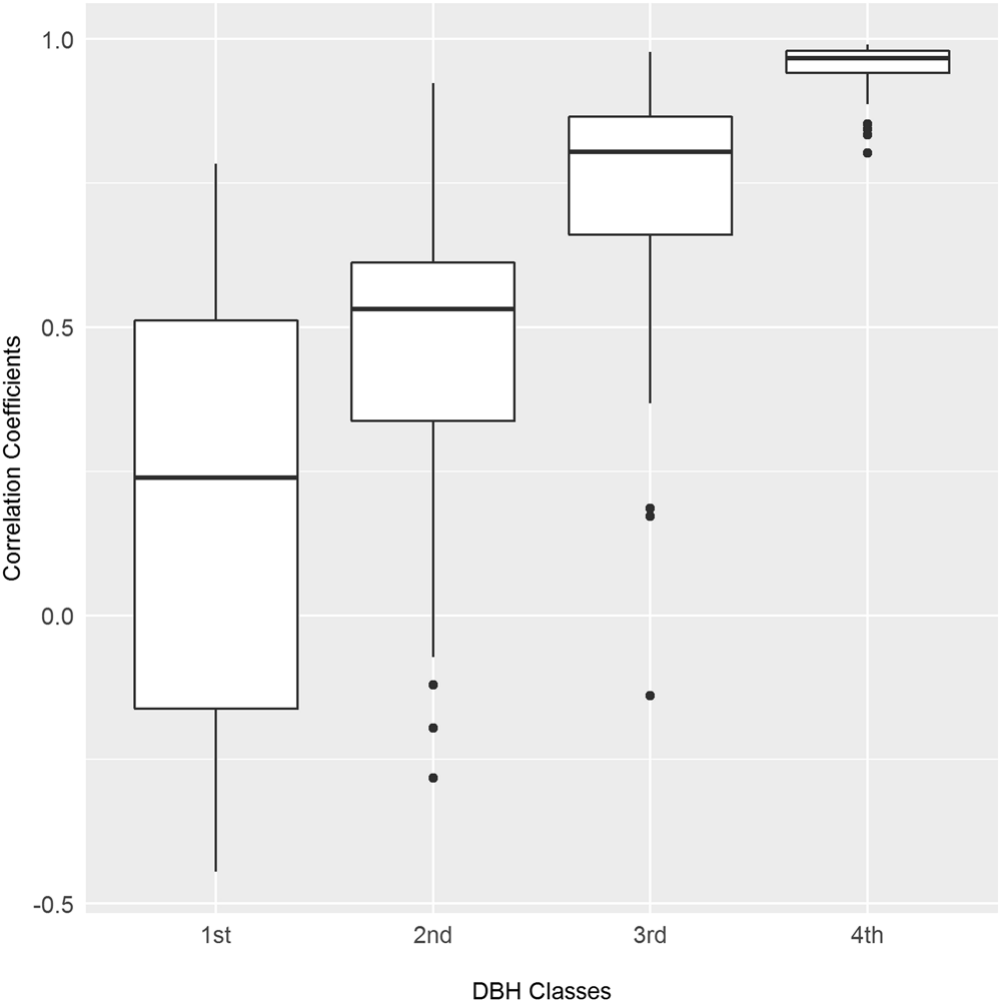
Correlations of plot NPP with summed NPP series for trees within four DBH class. Trees in each plots were grouped into four quartiles (4^th^ for the 1/4 largest trees) based on their DBH. Each box represents the variation of correlation coefficients for 36 plots.

Figure 2 showed that, the correlation between plot NPP series and the NPPs in each DBH class increased remarkably with tree size (median *r* = 0.238, 0.531, 0.804 and 0.967 for the 1^st^, 2^nd^, 3^rd^ and 4^th^ DBH class, respectively). This suggests that the annual dynamic of stand NPP is also mainly driven by large trees (supporting our P2).

**Figure 3:**
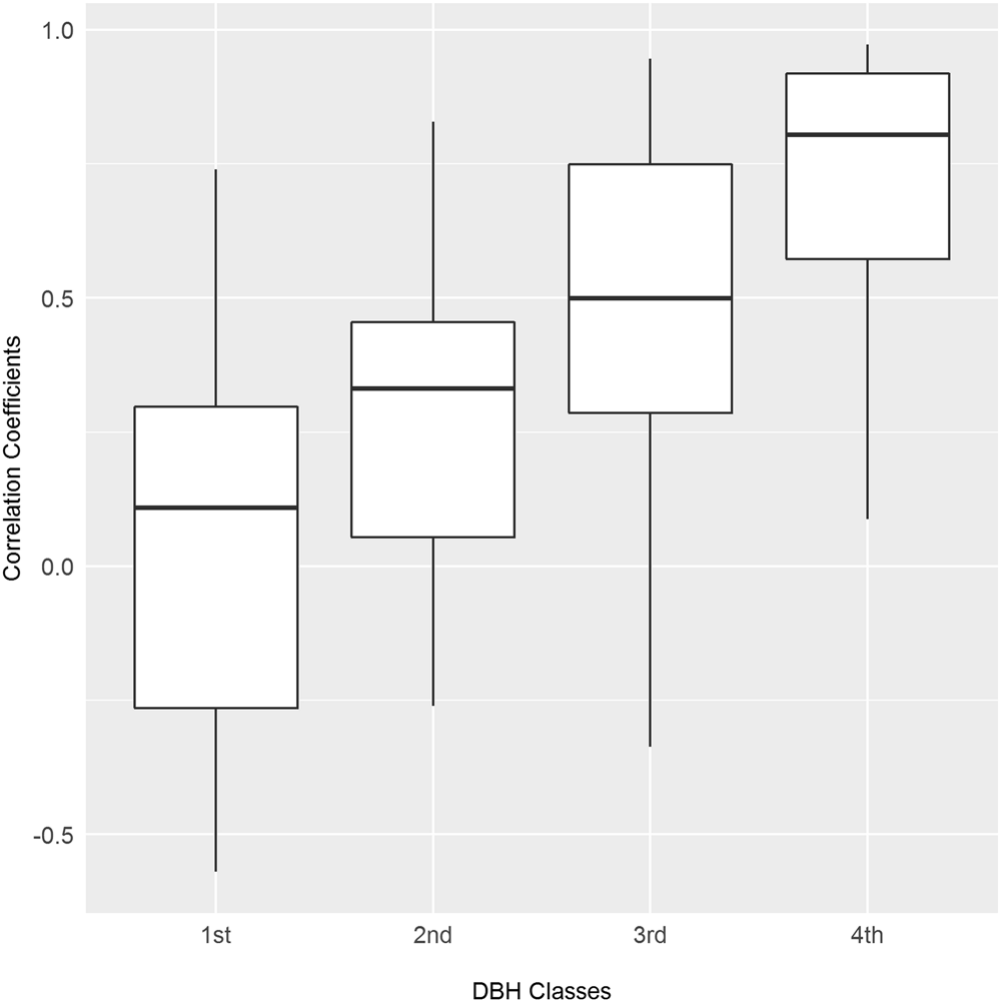
Correlations of RWI with tree NPP series of four DBH classes within each plot. Each box represents the variation of correlation coefficients for 36 plots.

Similarly, RWI was more closely related to annual NPP of the largest 1/4 trees (Fig. 3), but the correlation decreased with smaller tree size (median *r* = 0.109, 0.331, 0.499 and 0.804 for the 1^st^, 2^nd^, 3^rd^ and 4^th^ DBH class, respectively). Thus, our P3 was also supported.

## Discussion

### Tree-ring widths are good proxies for annual NPP variation at stand level

In this analysis, we used data along a latitudinal gradient to test whether RWI can be used as proxy for temporal variation in stand NPP (Question 1). Our results showed that, in most cases, RWI could well reflect annual NPP dynamic at stand level (Fig. 1, Table 2).

As for why RWI can reflect the annual variability of stand NPP (Question 3), we proposed a hypothesis including two parts: (1) stand NPP is large determined by the temporal variation in NPP of large trees; (2) the latter is closely related to annual DBH growth of large trees. Recent studies have increasingly recognized the critical roles of large trees in forest ecosystems. In addition to recent evidence that stand biomass is mainly occupied by large canopy trees^22–24^, here we further showed that stand NPP and its annual variation were also mainly determined by large trees (Table 3, Fig. 2; supporting our P1 and P2). Thus large trees act as major contributors of not only carbon pools but also carbon sinks in forest communities. Large trees are vulnerable to climate change, especially to the drought caused by climatic warming^22, 23, 35^. This is because the growth and mortality of large trees are more sensitive to water deficit than smaller trees as a result of their high stature (which lead to greater difficulty in vertical water transportation to canopy). Northeast China has experienced the most drastic climate warming in China and the related drought trends is also evident in the past decades^36^, which have showed clear negative impacts on forest growth^37^. Our results suggest the need for further examining the role of large trees in forest dynamics, and the conservation of large trees in response to climate change.

The second part of our hypothesis (P3) was also supported by our results that RWI were closely related to the NPP series of large trees instead of small ones (Fig. 3). Studies that used RWI to calibrate biogeochemistry models have long worried that how stand NPP is allocated to DBH growth is still not well understood^17^. Our results suggest that at least for large canopy trees DBH increment is proportional to annual NPP. This close relationship can be explained as follows. (1) It is well known that the proportion of stem biomass increase with tree size, and for large trees stem biomass can account for up to 76% of total biomass^38^. Meanwhile, theory predicted that the NPP of different tree organs is proportional to biomass of the organs^25^. Thus, the annual aboveground NPP variations of large trees should be mainly determined by stem NPP. (2) The proportion of stem NPP allocated to height vs. DBH growth also decrease with tree size, and the DBH growth of large trees will continue for many years after their heights have reach the asymptote^39–41^. This means that for large trees stem NPP is mainly allocated to DBH growth, thus it is logical that the annual NPP and RWI of large trees are highly correlated.

In summary, through testing our hypothesis, we showed that the close relationship between stand NPP and RWI series has sound biological basis. Our results support the use of RWI to validate process-based models, depending on that tree-ring chronologies are developed appropriately (see below).

### Which type of chronology is a better surrogate of stand NPP (Question 2)

Among the three chronologies commonly used in dendrochronology, our results reveal that standard or arstan chronologies are good proxies of stand NPP series while residual chronology is not (Table 2). This difference may be caused by the difference in the growth signals retained in residual vs. the other two chronologies. In standard chronology, the autocorrelation unique to each tree-ring series is retained, while in the residual chronology all autocorrelation is removed through autoregressive modelling. However, climate often exhibits year-to-year persistence that generates low-frequency trends in tree growth^42^, and removal of all autocorrelation may also remove the persistence common to all tree-ring series^43–45^. Thus in arstan chronology the common persistence was re-incorporated^20^. In our study, stand NPP is calculated as the sum of individual NPP series, with the common persistence included. This may be an important reason why the correlation between RWI and stand NPP is generally lower for residual chronology. Cook^20^ have pointed out that arstan chronology may have only marginally improve the time-series characteristics compared with standard chronology; our results is consistent with this idea because these two chronologies did not show marked difference in their correlations with stand NPP series.

### Suggestions for future research

While our results showed that RWI could well reflect the annual variation of stand NPP in most cases, there were still one exception. Examine why this exception happened is important if we want to use RWI as a reliable data source to validate biogeochemistry models. The species richness of DBF at Mt. Changbai is remarkably higher than that of other three sites because of favorable climate. The construction of tree-ring chronology including multiple species has proven difficult, because different species can show quite different inter-annual variability within a same stand (e.g. refs 46 and 47). For the DBF of Mt. Changbai, we also found it impossible to construct a chronology including all dominant species. Consequently, the final chronologies included cores mainly from *Acer mandshuricum*, *Syringa reticulate and Ulmus laciniata*, while other dominant species (e.g. *Tilia* spp.) were not represented. However, stand NPP series were built based on trees from all species, thus the mismatch (between NPP and RWI) was caused by the fact that the annual growth variation of some dominant species was not reflected in the chronology. The building of multi-species chronologies may be even difficult in ecosystems that are more species-rich. For instance, in subtropical forests there are a number of species co-exist in canopy layer with no species with clear dominance, and studies have found that different species showed a large variety of radial growth response to climate change (e.g. ref. 48). For these ecosystems, we suggest that whether RWI can be used as proxy for stand NPP still need careful tests, before RWI can be used for model validation.

In summary, our results showed that RWI can be used as a good proxy of annual stand NPP to validate biogeochemistry models in most cases, and that standard and arstan instead of residual chronology were preferred for this purpose. However, the exception found in this study also revealed that not all RWI can be safely used for model calibration. We have two suggestions for future studies that utilize RWI for model calibration: (1) the largest trees have predominant influence on annual variation of stand NPP, and thus should be sampled whenever possible; (2) dominant species in the canopy layer, especially the large individuals of them, should be included in tree-ring sampling and chronology construction. If these criterions were not meet, the chronologies may cannot be used for model validation. Our study area covered most of the latitudinal range of temperate forests in northeast China, thus these results ought to be appropriate for temperate forests in other regions, and probably can also apply to boreal forests (which are more species-poor). For (sub)tropical forests with diverse canopy species, however, we suggest that more tests are still needed before drawing a conclusion.

## Electronic supplementary material

Supplementary Information
