# Supplementary material for: Tree-ring widths are good proxies of annual variation in forest productivity in temperate forests
Source: Sci Rep. 2017 May 16;7:1945. doi: 10.1038/s41598-017-02022-6 (PMC5434002; doi:10.1038/s41598-017-02022-6)
Supplement: Supplementary file 1 — Supplementary Information [file 41598_2017_2022_MOESM1_ESM.pdf]

Tree-ring widths are good proxies of annual variation in forest productivity in temperate forests

Kai Xu, Xiangping Wang, Penghong Liang, Hailong An, Han Sun, Wei Han and Qiaoyan Li

Supplementary Table S1 Chronologies statistics of three forest types at four study sites. Abbreviations: MS, mean sensitivity; SD, standard deviation; AC, autocorrelation coefficient; SNR, signal-to-noise ratio; EPS, expressed population signal; BPF, *Betula* & *Populus* forest; DBF, deciduous broad-leaved forest; MBNF: mixed broad- & needle-leaved forest; STD, standard chronology; RES, residual chronology; ARS, arstan chronology.

Site	Forest Type	Chronology	MS	SD	AC	SNR	EPS
Mt. Changbai	BPF	STD	0.170	0.170	0.244	11.586	0.890
		RES	0.184	0.151	-0.135	12.105	0.902
		ARS	0.159	0.160	0.256	-	-
	DBF	STD	0.195	0.264	0.495	5.394	0.818
		RES	0.216	0.199	-0.025	5.868	0.846
		ARS	0.180	0.240	0.240	-	-
	MBNF	STD	0.169	0.211	0.568	5.654	0.850
		RES	0.197	0.158	-0.126	9.128	0.901
		ARS	0.155	0.170	0.379	-	-

		STD	0.231	0.204	0.060	19.354	0.945
	BPF	RES	0.195	0.173	-0.003	16.621	0.941
		ARS	0.228	0.223	0.314	-	-
		STD	0.197	0.268	0.594	10.116	0.908
Jiaohe	DBF	RES	0.229	0.196	-0.001	7.348	0.866
		ARS	0.189	0.266	0.594	-	-
		STD	0.181	0.550	0.750	8.866	0.884
	MBNF	RES	0.221	0.217	-0.036	7.549	0.882
		ARS	0.182	0.341	0.718	-	-
		STD	0.197	0.242	0.514	12.319	0.902
	BPF	RES	0.215	0.194	0.024	11.967	0.913
		ARS	0.185	0.211	0.39	-	-
		STD	0.169	0.227	0.476	9.668	0.930
Wuying	DBF	RES	0.178	0.181	0.042	8.725	0.913
		ARS	0.156	0.200	0.412	-	-
		STD	0.164	0.227	0.578	7.246	0.875
	MBNF	RES	0.180	0.158	-0.016	14.156	0.934
		ARS	0.156	0.183	0.374	-	-
		STD	0.259	0.385	0.618	23.748	0.958
	BPF	RES	0.294	0.286	0.197	16.243	0.941
		ARS	0.259	0.355	0.529	-	-
		STD	0.223	0.263	0.499	9.635	0.906
Shengshan	DBF	RES	0.253	0.219	-0.031	7.557	0.881
		ARS	0.208	0.254	0.472	-	-
		STD	0.197	0.430	0.841	10.941	0.914
	MBNF	RES	0.209	0.208	0.107	6.586	0.867
		ARS	0.173	0.325	0.656	-	-

Supplementary Table S2 The period and the tree species included in the chronology of each forest type at four study sites. Abbreviations: BPF, *Betula* & *Populus* forest; DBF, deciduous broad-leaved forest; MBNF: mixed broad- & needle-leaved forest.

Site	Forest Type	Period (yr.)	Specie
Mt. Changbai	BPF	1953-2012	*Betula platyphylla*<br>*Quercus mongolica*<br>*Fraxinus mandschurica*<br>Others
	DBF	1769-2012	*Acer mandshuricum*<br>*Syringa reticulata*<br>*Ulmus laciniata*<br>*Quercus mongolica*<br>Others
	MBNF	1861-2012	*Pinus koraiensis*<br>Others
Jiaohe	BPF	1967-2012	*Betula platyphylla*<br>*Populus cathayana*<br>*Ulmus davidiana*
	DBF	1771-2012	*Acer mono*<br>*Pinus koraiensis*<br>Others
	MBNF	1869-2012	*Pinus koraiensis*<br>*Fraxinus mandschurica*<br>Others
Wuying	BPF	1839-2013	*Betula platyphylla*<br>Others
	DBF	1941-2013	*Fraxinus mandschurica*<br>*Padus maackii*<br>*Betula costata*<br>*Betula platyphylla*<br>*Populus davidiana*<br>Others

	MBNF	1760-2013	*Pinus koraiensis*<br>Others
	BPF	1951-2013	*Betula platyphylla*<br>Others
Shengshan	DBF	1893-2013	*Quercus mongolica*<br>*Betula dahurica*
	MBNF	1805-2013	*Pinus koraiensis*<br>*Picea koraiensis*<br>Others
